# A Psychometric Examination of the Coronavirus Anxiety Scale and the Fear of Coronavirus Disease 2019 Scale in the Italian Population

**DOI:** 10.3389/fpsyg.2021.669384

**Published:** 2021-06-18

**Authors:** Graziella Orrù, Davide Bertelloni, Francesca Diolaiuti, Ciro Conversano, Rebecca Ciacchini, Angelo Gemignani

**Affiliations:** Department of Surgical, Medical and Molecular Pathology and Critical Care Medicine, University of Pisa, Pisa, Italy

**Keywords:** COVID-19 pandemic, anxiety, fear, CAS, FCS-19S, mental health, psychopathology

## Abstract

The coronavirus disease 2019 (COVID-19) outbreak has caused not only significant physical health problems but also mental health disorders. Anxiety and fear appear to be the main psychological symptoms associated with COVID-19. The aim of this study was to investigate whether anxiety and fear related to COVID-19 are influenced by sociodemographics and whether specific conditions, such as positivity for COVID-19 or death among relatives and friends, can further enhance these symptoms. In this cross-sectional study, 697 Italians responded to an online survey assessing sociodemographic information, the presence/absence of positive cases, or deaths due to COVID-19 among relatives or acquaintances. The Coronavirus Anxiety Scale (CAS) and Fear of COVID-19 Scale (FCS-19S) were administered in order to assess the levels of anxiety and fear associated with COVID-19. The data were collected in November 2020. Anxiety and fear scores were positively correlated. Both male and female subjects with higher CAS scores also displayed higher FCS-19S scores. The CAS and FCS-19S scores tended to increase with age, with older subjects exhibiting higher scores than younger subjects. Additionally, respondents with lower educational levels demonstrated higher scores on both the CAS and FCS-19S. Similarly, respondents living with older people and/or experiencing the death of one or more relatives due to COVID-19 exhibited corresponding outcomes. This study demonstrates how the levels of anxiety and fear, measured by CAS and FCS-19S associated with COVID-19, are influenced by gender, age, cohabitation status, educational levels, and the presence of positive cases or deaths due to COVID-19.

## Introduction

The coronavirus disease 2019 (COVID-19) was declared a worldwide pandemic by the World Health Organization (WHO) in March 2020, but its outbreak was first documented in Wuhan, China, in December 2019. The number of confirmed cases stands at more than 110 million worldwide, while the COVID-19 death toll has passed 2 million (www.worldometers.info; accessed on February 17, 2020 at 03.24 p.m.).

At the time of writing, the COVID-19 pandemic outbreak is undoubtedly causing, and will continue to cause, disastrous economic, financial, political, social, and psychological consequences. While the initial focus was directed toward the physical manifestations of infection (i.e., pneumonia, fever, cough, fatigue, slight dyspnea, headache, and sore throat) (Pascarella et al., 2020), the scientific community soon identified the negative impact of COVID-19 on the brain (Orrù et al., 2020), and mental health (Brooks et al., 2020; Di Giuseppe et al., 2020; Iachini et al., 2020; Orrù et al., 2021). A significant additional factor should be noted in terms of the impact of COVID-19 on emerging adults, afraid not only of the possible negative effects of COVID-19 personally but also in terms of their role as possible carriers in the face of weaker and more fragile subjects. A study carried out by Germani et al. (2020) demonstrated that young people are more worried and afraid of COVID-19 than those “older” than themselves, precisely because of the greater possibility of becoming potential symptomatic or asymptomatic carriers (Germani et al., 2020). The Inter-Agency Standing Committee Guidelines on Mental Health and Psychosocial Support recommends the integration of multiple levels of intervention in response to epidemics and/or emergency situations. In this context, researchers and clinical psychologists attempt to address such consequences by accurately screening the psychological and psychosocial impact of COVID-19, by planning specific tailored interventions accordingly. Researchers also attempt to identify techniques aimed at the prevention of the development of psychological distress due to COVID-19, such as the investigation of Lopez et al. on the role of mindfulness before and during the COVID-19 lockdown, revealing that COVID-19-related anxiety and fear interfere with the mindfulness profile (Lopez et al., 2021).

In fact, a significant increase in psychological distress and associated disorders (i.e., generalized anxiety, panic disorder, and acute stress disorder) has been observed in the general population worldwide over the last 8 months (Iachini et al., 2020; Qian and Li, 2020). Specifically, Iachini et al. (2020) investigated the psychological factors regulating the influence of interpersonal space (such as the 1.80 m of interpersonal distance required by law) and its impact on psychological well-being, finding that higher levels of anxiety, fear, and stress have led to an increase in interpersonal distances, causing a significant decrease in psychological well-being. More specifically, the abovementioned conditions caused by the spread of COVID-19 create not only uncertainty, fear, denial, anxiety, insomnia, dissociative symptoms, depressive disorders, emotional distress, suicidal thoughts or attempts to commit suicide, alcohol, and substance misuse in the general population but also the risk of relapse in those living with preexisting mental disorders (Di Giuseppe et al., 2020; Torales et al., 2020). Such factors are also particularly relevant to healthcare professionals as they represent worsening factors both in terms of their own mental health and their professional performance in areas such as the care plan treatment of patients (Xiang et al., 2020) and communication with family members during the final stage of life of an individual (Iasevoli et al., 2012).

Negative emotions such as anxiety and fear may be related to a number of aspects of the COVID-19 pandemic, specifically physical deterioration, prolonged distress, the death of a loved one, social isolation or prolonged confinement, financial hardship, and uncertainty (Huang and Zhao, 2020). Indeed, the study carried out by Huang and Zhao (2020) during the COVID-19 outbreak demonstrated that the overall rate of anxiety in the general population was approximately 35.1%. This outcome is particularly significant and indicative of the importance of prioritizing psychological testing aimed at an in-depth understanding of signs and symptoms (Orru et al., 2009), as well as offering adequate procedures and prompt responses.

The purpose of this study was to investigate the levels of fear and anxiety measured by using the Coronavirus Anxiety Scale (CAS) (Mozzoni and Franzot, 2020; Sherman and Lee, 2020) and the Fear of COVID-19 Scale (FCS-19S) (Ahorsu et al., 2020; Soraci et al., 2020), associated with the COVID-19 outbreak in the Italian population. Specifically, the investigation was carried out as to whether those domains were influenced by variables such as gender, age, cohabitation status, and educational levels. Additionally, this study explored whether specific factors such as positivity for COVID-19 or death among friends, relatives, and acquaintances may further enhance the levels of fear and anxiety.

In accordance with the abovementioned aims, two validated measures, namely, CAS and FCS-19S, were administered.

## Materials and Methods

### Participants

A total of 704 responses were gathered through an online survey conducted between November 1 and November 10, 2020 from individuals living in Italy. Of these, seven were excluded from subsequent analyses (i.e., one did not fall within the target age range and six did not provide the informed consent). A total of 697 participants were thus assigned to final analyses (i.e., *n* = 107 males; *n* = 590 females).

### Instruments

The subjects were asked specific COVID-19-related information such as the presence/absence of positive cases or deaths among relatives, close friends, or acquaintances. The CAS (Mozzoni and Franzot, 2020; Sherman and Lee, 2020) and FCS-19S (Ahorsu et al., 2020; Soraci et al., 2020) were employed in order to assess the levels of fear and anxiety due to COVID-19.

The CAS (Mozzoni and Franzot, 2020; Sherman and Lee, 2020): The CAS is a self-report tool designed to measure the levels of dysfunctional anxiety associated with COVID-19. It is made up of five items employing a 5-point Likert scale (i.e., responses ranging from *never* to *almost every day in the last 2 weeks*). Should the total score equal 9 or higher, dysfunctional anxiety is likely.

Fear of COVID-19 Scale (Ahorsu et al., 2020; Soraci et al., 2020): The FCS-19S is a self-report tool designed to measure the levels of dysfunctional fear associated with COVID-19. It is made up of five items employing a 5-point Likert scale (responses ranging from *strongly disagree* to *strongly agree*). The minimum score possible for each question is 1 and the maximum is 5. The total score can be calculated by adding the score of each item (ranging from 7 to 35).

### Procedures

The survey was launched online on November 1, 2020 at 9:00 a.m. (GMT + 1). Respondents were recruited through social media campaigns on sites including Facebook and LinkedIn or through a direct link sent to participants by e-mail. The participants were informed of the purposes of the study and were asked to provide consent on the treatment of personal data. All procedures followed the ethical standards and were approved by the Ethics Committee of the University of Pisa (No. 0036344/2020).

### Statistical Analysis

Descriptive statistics were used in order to examine the available data. For each variable, the number of subjects and the relative percentage of the total scores, as well as the mean (*M*) and *SD* of the scores obtained through CAS and FCS-19, were reported. Comparisons between the female and male groups were performed using a *t*-test. The Spearman's correlation was calculated in order to assess the relationships between psychological variables, sociodemographic characteristics, and COVID-19 situational experiences. The *p*-values <0.05 and 95% confidence intervals were considered statistically significant. The significant correlations were then further investigated. The analysis was conducted using R-3.4.3 for Windows.

## Results

A total of 697 participants (*n* = 107 males; *n* = 590 females) completed the online survey. The sociodemographic characteristics of the sample, measurement scores, and COVID-19 experiences are summarized in Table 1.

**Table 1:** Sample characteristics describe the number of subjects (*N*), percentage (%), mean (*M*), and standard deviation (*SD*).

	***N* = 697**	***N* (%)**	**CAS and FCS-19S scores**			
	**Features**					
			**CAS score**		**FCS-19S score**	
		***N*/%**	***M***	***SD***	***M***	***SD***
Sex	Male	107 (15.35)	4.59	4.25	19.05	6.02
	Female	590 (84.65)	7.01	5.37	7.13	7.13
	All subjects	697 (100)	4.96	4.52	6.31	6.31
Age	<20	32 (4.59)	3.41	2.21	18.69	5.23
	20–29	273 (39.17)	3.69	3.44	17.84	5.58
	30–39	125 (17.93)	4.35	4.00	18.50	5.74
	40–49	111 (15.93)	6.22	5.11	21.01	6.39
	50–59	107 (15.35)	7.40	5.65	23.05	7.31
	60–69	44 (6.31)	6.36	4.66	21.43	5.43
	>70	5 (0.72)	7.20	6.91	22.4	7.13
Residence	Northern Italy	265 (38.02)	3.47	3.27	17.57	5.58
	Center of Italy	299 (42.90)	5.54	4.86	20.20	6.46
	Southern Italy and islands	118 (16.93)	6.91	5.02	22.45	6.10
	Other	15 (2.15)	4.60	4.52	19.33	6.17
Cohabitation status	Living alone	612 (87.80)	4.74	4.39	19.24	6.17
	Living with one or more relatives	87 (12.48)	6.55	5.11	21.91	6.82
	Not living with older people	581 (83.36)	4.53	4.20	18.99	6.00
	Living with older people	118 (16.93)	7.15	5.36	22.43	7.03
Educational Level	Middle School (8 years)	37 (5.31)	2.57	2.13	17.12	5.10
	High School (13 years)	272 (39.02)	3.72	3.39	17.69	5.56
	Three-year degree (16 years)	174 (24.96)	6.15	5.36	21.03	6.90
	Master's degree (18 years)	144 (20.96)	5.88	4.60	20.97	6.07
	Master's degree (17–19 years)	42 (6.03)	7.07	5.33	22.19	6.62
	Ph.D. (22 years)	18 (2.58)	5.83	5.43	21.22	6.61
	Missing data	10 (1.43)	3.40	2.50	19.40	4.62
Positivity to COVID-19	0	498 (71.45)	4.48	4.29	18.85	6.22
	1	88 (12.63)	5.50	4.26	20.64	5.99
	2	57 (8.18)	7.37	5.63	22.30	6.69
	>3	54 (7.75)	6.00	4.77	21.48	5.91
Death due to COVID-19	0	586 (84.05)	4.63	4.29	19.12	6.22
	>1	108 (15.49)	6.75	5.28	21.92	6.33
	Missing data	3 (0.43)				

*Notes: CAS and FCS-19S scores stratified according to sex, age, cohabitation status, educational levels, positive cases for COVID-19, and deaths caused by COVID-19. CAS, Coronavirus Anxiety Scale; FCS-19S, Fear of COVID-19 Scale; COVID-19, coronavirus disease 2019*.

The correlation analysis between demographics, CAS, FCS-19S, and COVID-19 situational experiences is reported in Table 2. A positive correlation was identified in the case of age and CAS score (*r* = 0.30) and of CAS and FCS-19S scores (*r* = 0.76).

**Table 2:** Spearman's correlation coefficient (*r*) among CAS, FCS-19S, demographics, and COVID-19 situational experiences.

**Features**	**1**	**2**	**3**	**4**	**5**	**6**	**7**	**8**
1. Sex	1.00	0.05	0.02	0.02	−0.06	0.14	−0.13	−0.16
2. Age	0.05	1.00	−0.15	−0.17	0.22	0.01	0.30	0.28
3. Cohabitation status	0.02	−0.15	1.00	0.05	−0.02	0.01	−0.03	−0.06
4. Educational Level	0.02	−0.17	0.05	1.00	−0.12	0.07	−0.10	−0.14
5. Positivity to COVID-19	−0.06	0.22	−0.02	−0.12	1.00	0.22	0.15	0.14
6. Deaths due to COVID-19	0.14	0.01	0.01	0.07	0.22	1.00	0.12	0.13
7. CAS Score	−0.13	**0.30**	−0.03	−0.10	0.15	0.12	1.00	0.76
8. FCS-19S Score	−0.16	0.28	−0.06	−0.14	0.14	0.13	**0.76**	1.00

*Notes: The values in bold are different from 0 at significance level of α = 0.05. CAS, Coronavirus Anxiety Scale; FCS-19S, Fear of COVID-19 Scale; COVID-19, coronavirus disease 2019*.

With regard to the CAS, a score of ≥9 indicated a level of anxiety beyond the normal levels. In this study, 141 subjects displayed scores of ≥9, approximately 20.23% of the total sample. No cut-offs are available in the case of the FCS-19S. However, the questionnaire indicates that the scores range from 5 to 35 with higher scores suggesting higher levels of fear. A total of 108 subjects (i.e., 15.49% of total participants) showed scores between 25 and 30, while 51 subjects (i.e., 7.32% of total participants) scored >30. Overall, 159 subjects (i.e., 22.81% of total participants) reported the FCS-19S scores of >25.

### CAS and FCS-19S Scores and Gender Differences

**Figure 1:**
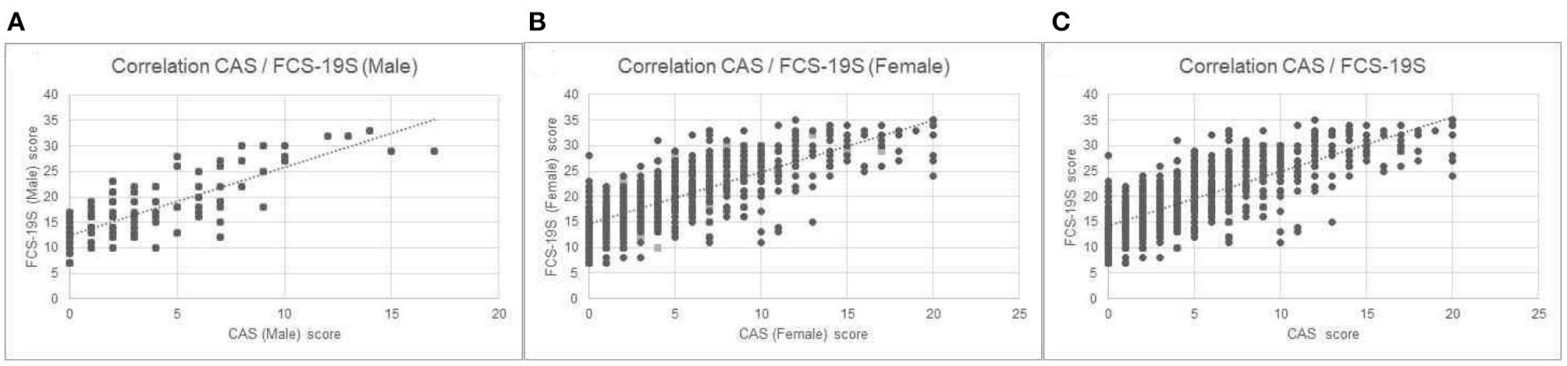
**(A)** Correlation between CAS and FCS-19S scores; **(B)** correlation between CAS scores and FCS-19S scores in the female subgroup; and **(C)** correlation between CAS scores and FCS-19S scores in the male subgroup. CAS, Coronavirus Anxiety Scale; FCS-19S, Fear of COVID-19 Scale; COVID-19, coronavirus disease 2019.

The correlation analysis demonstrated a significant gender difference in CAS and FCS-19S scores (Figure 1). Specifically, the data analysis displayed higher CAS and FCS-19S scores in females than in males (*p* < 0.001). The data also revealed a positive and significant correlation between CAS and FCS-19S scores. In fact, subjects with higher scores on the CAS also displayed higher scores on the FCS-19S (*r* = 0.76; *p* < 0.05) (Figure 1A). Such correlations were also observed in the subgroups composed of males (*r* = 0.74; *p* < 0.05) and females (*r* = 0.81; *p* < 0.05) (Figures 1B,C, respectively).

### CAS and FCS-19S Scores and Age

**Figure 2:**
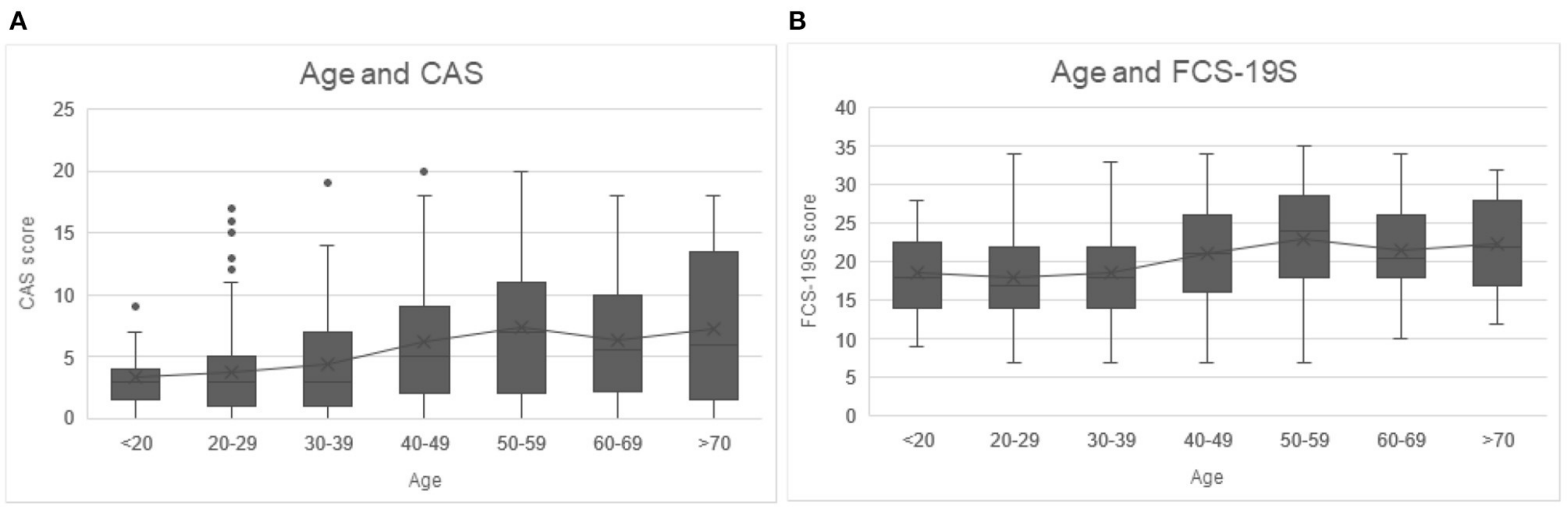
**(A)** CSA scores increased as people age, with higher CAS scores in the age group ranging from 50 to 59 and >70. **(B)** Higher FCS-19S scores as people age, with increasing age of the subjects, with higher FCS-19S scores in the age group ranging from 50 to 59 years and >70. CAS, Coronavirus Anxiety Scale; FCS-19S, Fear of COVID-19 Scale; COVID-19, coronavirus disease 2019.

A positive correlation was identified for age, CAS, and FCS-19S (see Table 1), and age and CAS score (*r* = 0.30) (see Table 2). As it is evident from Figures 2A,B, the CAS and FCS-19S scores increased with age. Specifically, age groups with the highest scores were those ranging from 50 to 59 and >70, both for CAS and FCS-19S.

### CAS and FCS-19S Scores and Cohabit Status

No significant differences were detected between CAS and FDS-19S scores in people living alone compared with those living with one or more relatives (i.e., children and spouse). However, subjects living with elderly people showed significantly higher levels of anxiety (i.e., higher CAS scores) than those who do not (*p* < 0.01). Similarly, higher levels of fear (i.e., higher FDS-19S scores) were observed in subjects living with elderly people compared with those who do not, yet no significant differences were identified (*p* = 0.058).

### Does the Educational Level Influence CAS and FCS-19S Scores?

**Figure 3:**
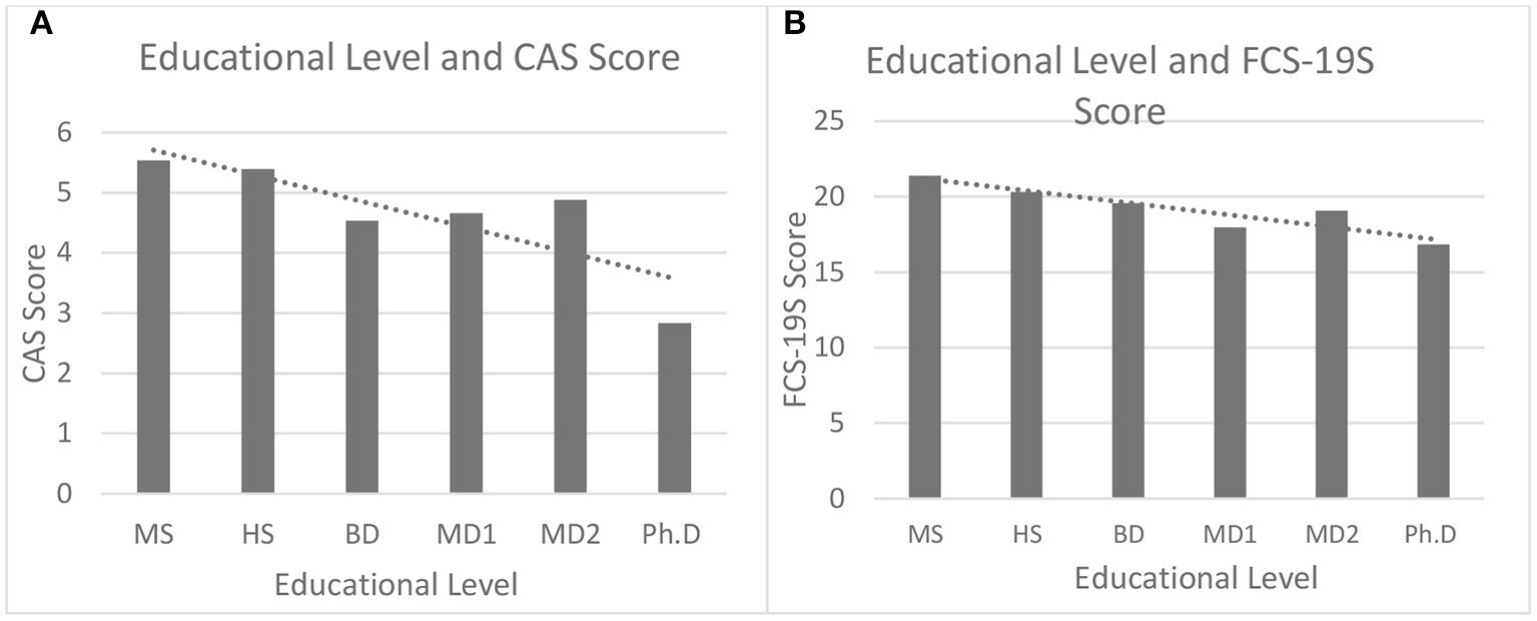
The relationship between the level of education and CAS scores **(A)** and FCS-19S scores **(B)**. MS, Middle School; HS, High School; BD, Bachelor's Degree; MD1, Master's Degree; MD2, 1st or 2nd level Master's Degree; PhD, PhD.; CAS, Coronavirus Anxiety Scale; FCS-19S, Fear of COVID-19 Scale; COVID-19, coronavirus disease 2019.

This study also investigated whether educational levels can be associated with CAS and FCS-19S scores or, rather, whether higher levels of education can be associated with lower CAS and FDS-19S scores. The sample was originally gathered according to the following levels of education: (i) Middle School (MS) (8 years); (ii) High School (HS) (13 years); (iii) Bachelor's Degree (BD) (16 years); (iv) Master's Degree (MD1) (18 years); (v) 1st or 2nd level Master's Degree (MD2) (1st level MD2: 17 years if obtained subsequent to BD; 2nd level MD2: 19 years if obtained subsequent to MD1); and (vi) Ph.D. (Ph.D.) (22 years). Figure 3 illustrates the relationship between the educational levels and scale scores measured through CAS and FCS-19S. Higher levels of education were associated with lower CAS and FCS-19S scores.

**Figure 4:**
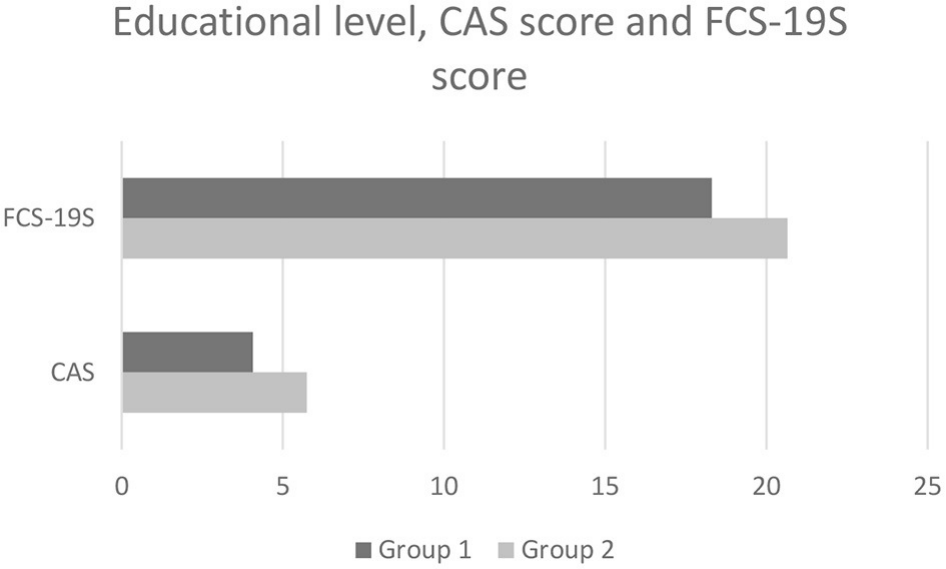
The average CAS and FCS-19S scores in Group 1 vs. Group 2. CAS, Coronavirus Anxiety Scale; FCS-19S, Fear of COVID-19 Scale; COVID-19, coronavirus disease 2019.

For a better understanding of the effects relating to higher and lower educational levels on CAS and FCS-19S scores, the participants were divided into two subgroups based on the educational levels: the first subgroup (Group 1) included individuals with MS and HS qualifications, while the second subgroup (Group 2) included individuals with BD, MD (1 and 2), and Ph.D. qualifications. Significantly higher CAS (*p* < 0.01) and FCS-19S scores (*p* < 0.01) were detected in Group 1 as compared with Group 2 (Figure 4). These data suggest that individuals with a lower educational level may be more susceptible to experiencing higher levels of anxiety and fear.

### CAS and FCS-19S Scores and Positivity for COVID-19

In order to investigate whether specific conditions such as the presence/absence of COVID-19 positivity or COVID-19-related deaths among friends and/or relatives may further enhance the levels of fear or anxiety measured by CAS and FCS-19S, every individual was asked to reply to the following questions:

- How many cases of COVID-19 positivity (including yourself) can you report among your family members?
- How many deaths have you suffered due to COVID-19 among family members or acquaintances?

**Figure 5:**
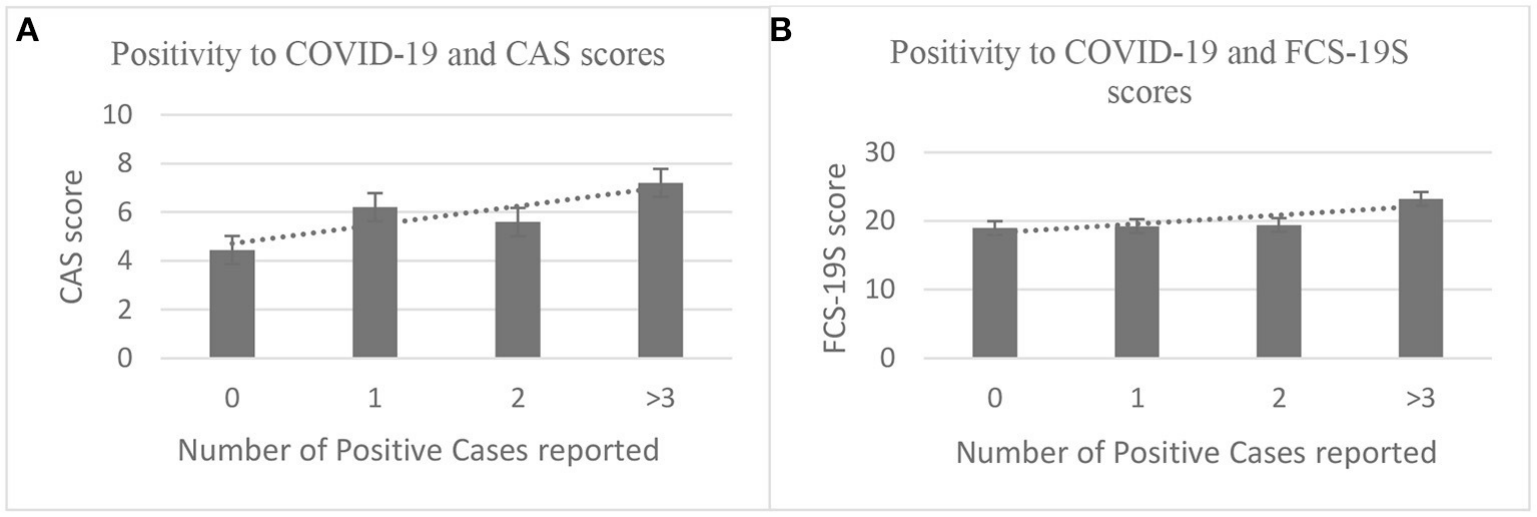
Higher CAS and FCS-19S scores were associated with a higher number of positive cases reported [**(A, B)**, respectively]. CAS, Coronavirus Anxiety Scale; FCS-19S, Fear of COVID-19 Scale; COVID-19, coronavirus disease 2019.

In terms of the first question, the results showed those subjects indicating at least one case of positivity to COVID-19 among family members displayed significantly higher CAS (*p* < 0.01) and FCS-19S scores (*p* < 0.01) compared with those who did not indicate any positive cases of COVID-19. The results for the second question illustrated that individuals who have experienced the death of at least one family member due to COVID-19 also displayed higher CAS and FCS-19S scores than those who had not (Figure 5).

**Figure 6:**
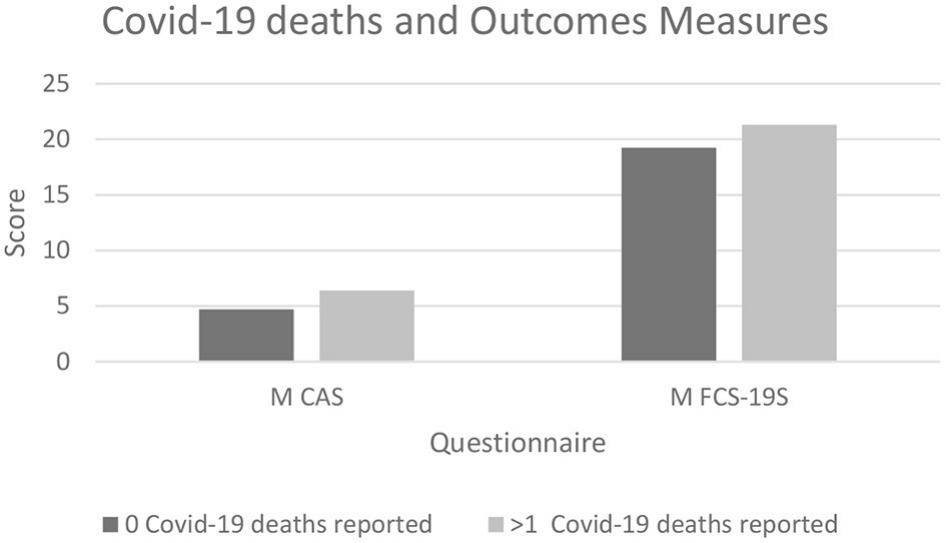
The graph shows significantly higher CAS and FCS-19S scores in subjects who have suffered at least one loss due to COVID-19 (*p* < 0.001 and *p* < 0.01, respectively) compared with those who did experience it. CAS, Coronavirus Anxiety Scale; FCS-19S, Fear of COVID-19 Scale; COVID-19, coronavirus disease 2019.

With regard to the second question, the analyzed responses revealed that subjects reporting one or more deaths due to COVID-19 showed higher CAS and FCS-19S scores (*p* < 0.001 and *p* < 0.01, respectively) compared with those who did not report any deaths among family members or acquaintances (Figure 6).

## Discussion and Conclusion

The aim of this study was to investigate the levels of fear and anxiety, as measured by CAS and FCS-19S, associated with the COVID-19 outbreak among the Italian population. Specifically, the study investigated whether those domains were influenced by variables such as (1) gender, (2) age, (3) cohabitation status, (4) educational levels, and (5) situational experiences related to COVID-19. Additionally, this study aimed to explore whether specific factors such as positivity for COVID-19 or death among friends, relatives, and acquaintances may further enhance the levels of fear and anxiety.

This study demonstrates a possible positive correlation among different variables considered, i.e., anxiety, fear, and COVID-19 outbreak experiences. The anxiety and fear scores measured by CAS and FCS-19S were positively correlated, with subjects demonstrating higher CAS scores also showing higher FCS-19S scores. Similar outcomes were detected in the analysis of the subgroups investigating gender differences. Females showed significantly higher levels of anxiety and fear than males. These data are consistent with other findings, suggesting that, typically, females show a higher predisposition to anxiety compared with males (Foot and Koszycki, 2004; Seo et al., 2017). In fact, women have been reported to display higher rates of affective disorders than men (Bebbington et al., 1998; Cyranowski et al., 2000; Bijl et al., 2002; Kessler, 2003; Leach et al., 2008). According to a number of studies, the lifetime prevalence of affective disorders is approximately double in female subjects as compared with their male counterparts (Bebbington et al., 1998; Cyranowski et al., 2000; Bijl et al., 2002; Kessler, 2003; Leach et al., 2008). However, these findings are in contrast with other studies reporting lower scores (Faravelli et al., 2013; Salk et al., 2017). Taken collectively, the implications of the studies described above suggest that the findings reported preclude robust conclusions and require further investigation.

Additionally, an investigation was carried out in order to determine whether the levels of anxiety and fear associated with COVID-19 are negatively influenced by age. The findings demonstrate that CAS and FCS-19S scores tend to increase with age; in fact, older subjects presented higher scores as compared with younger subjects. Such outcomes are in contrast with other studies reporting lower scores as age increases (Faravelli et al., 2013; Salk et al., 2017).

In terms of the third aim, cohabitation status was analyzed. The results demonstrated that respondents living with elderly people exhibited higher levels of anxiety and fear associated with COVID-19. In addition, the educational level (the fourth aim of this study) was an extremely significant variable as subjects with a lower educational level demonstrated higher levels of anxiety and fear associated with COVID-19 compared with subjects with a higher educational level. Such results seem to successfully confirm other findings, which clearly demonstrate that educational levels may influence the degree of anxiety and depression experienced and reduce manifestations of psychopathological symptoms (Bjelland et al., 2008; Chazelle et al., 2011). In contrast, other studies have concluded that participants with higher educational levels reported higher psychological distress than those with lower levels (Qiu et al., 2020; Wang et al., 2020). Further studies clarifying and expanding on this issue are therefore of particular interest.

Finally, this study investigated whether specific conditions such as positivity for COVID-19 or death among friends and/or relatives may further enhance such symptoms. In this context, the results demonstrated that subjects reporting at least one case of positivity to COVID-19 among family members displayed significantly higher CAS and FCS-19S scores compared with those who did not indicate a positive case of COVID-19. Furthermore, the results demonstrated that individuals who have experienced the death of at least one family member due to COVID-19 displayed higher CAS and FCS-19S scores than those who had not.

This study has the following limitations: (1) the sample under this study is not sufficiently broad or representative of the general population (i.e., this study was hindered by a significant gender imbalance). Future studies must therefore aim to expand the number of participants and ensure that it is representative of the general population; (2) recruitment channels (mainly the Internet) may lead to bias, and it is therefore necessary that future studies expand and equalize recruitment channels; (3) the data were collected from November 1, 2020 to November 10, 2020, a period in which Italian legislation divided regions by color, with each color imposing varying limitations to social life and mobility. For these reasons, at the time of completing the questionnaire, the subjects were living in different regions with varying degrees of limitations, which may also have affected anxiety and fear levels. It is therefore considered appropriate that future studies investigate subjects living under the same legislative conditions (i.e., limitations to social life and mobility); (4) the CAS was validated in the English language by Sherman and Lee (2020), but has not yet been entirely validated in its Italian form. An online Italian translation by Mozzoni and Franzot (2020) is available, yet published data does not exist on Italian norms and validation of the scale. Future studies should therefore make use of validated tools or a validated Italian version of the CAS, if available.

In conclusion, this study suggests that anxiety and fear associated with COVID-19 are influenced by gender differences, age, cohabitation status, and education levels. Additionally, positivity to COVID-19 and death caused by COVID-19 seem to have profound adverse effects on mental health, death distress, perceived risk, and happiness among the general population.

## Data Availability Statement

The raw data supporting the conclusions of this article will be made available by the authors, without undue reservation.

## Ethics Statement

The studies involving human participants were reviewed and approved by University of Pisa. The patients/participants provided their written informed consent to participate in this study.

## Author Contributions

GO, DB, and FD devised the main research topic, planned the online survey, carried out the analysis, conceived the conceptual ideas and proof outline, and drafted the manuscript. GO, DB, FD, CC, RC, and AG revised the manuscript critically. GO and CC provided an extensive revision of the final version of the manuscript. All the authors gave the final approval for the version to be published.

## Conflict of Interest

The authors declare that the research was conducted in the absence of any commercial or financial relationships that could be construed as a potential conflict of interest.
